# A Personalized, Rate‐of‐Rise (ROR)–Driven Model to Optimize Red Cell Exchange Interval in Chronic Sickle Cell Management

**DOI:** 10.1002/jca.70169

**Published:** 2026-08-16

**Authors:** Menatalla Nadim, Austin Choi, Nicholas Parisi, Yamac Akgun

**Affiliations:** ^1^ Department of Pathology and Laboratory Medicine University of Miami Miller School of Medicine Miami Florida USA

**Keywords:** hemoglobin S, personalized scheduling, rate of rise, red blood cell exchange, sickle cell disease

## Abstract

Chronic automated red blood cell exchange (RCE) is a cornerstone therapy for preventing sickle cell disease (SCD)‐related complications, particularly stroke and recurrent vaso‐occlusive crises. Current scheduling commonly relies on uniform empirical intervals of 3–6 weeks and does not account for substantial inter‐patient variability in hemoglobin S (HbS) rebound kinetics, potentially resulting in undertreatment of rapid rebounders and unnecessary procedures in slower rebounders. We hypothesized that each patient's personalized rate of rise (ROR) of HbS could be used to generate an individualized, risk‐adjusted RCE interval. We retrospectively analyzed adult patients with SCD receiving chronic outpatient RCE. The primary cohort comprised 18 patients with HbSS, while a single patient with HbSC was analyzed separately. HbS values from consecutive outpatient procedures were used to calculate an individual ROR (%HbS/day). A personalized scheduling formula was derived: *t*(interval) = *S*(risk) × (HbS(threshold) − HbS(post))/ROR(personal), where *S*(risk) represents an adjustable safety factor for patients at higher clinical risk. Individualized intervals differed markedly from uniform scheduling. Slow rebounders (< 0.5%/day) could potentially extend beyond 5 weeks, whereas rapid rebounders (> 1.0%/day) reached prespecified HbS thresholds within approximately 2–3 weeks. For most patients, the model recommended shorter intervals than those used, suggesting that under current scheduling many patients may exceed their HbS threshold between procedures. Because most patients would require shorter rather than longer intervals to maintain HbS below 30%, strict implementation of the model could increase program‐level blood utilization; therefore, its principal value lies in precision and resource reallocation rather than guaranteed net savings. A personalized ROR‐based scheduling framework integrates objective HbS kinetics with clinical risk stratification and may be most applicable to patients with stable, reproducible rebound kinetics. Prospective validation and integration into electronic health record systems are warranted to determine whether model‐guided scheduling improves clinical outcomes.

## 1 Introduction

Sickle cell disease (SCD) is the most common inherited hemoglobinopathy worldwide, affecting an estimated 7.7 million individuals globally and approximately 100 000 in the United States [1, 2, 3]. A single substitution in the HBB gene produces hemoglobin S (HbS), which polymerizes under deoxygenated conditions and drives chronic hemolysis, vaso‐occlusion, and progressive organ damage, including stroke, acute chest syndrome, and pulmonary hypertension [2, 4].

Chronic prophylactic red blood cell exchange (RCE) has emerged as a cornerstone therapy for preventing the most devastating complications of SCD, particularly cerebrovascular events. The STOP trial showed that chronic transfusion reduces first‐stroke risk by 92% in children with abnormal transcranial Doppler velocities, and STOP 2 demonstrated reversion to high‐risk status upon discontinuation [5]. The SIT trial extended these benefits to silent cerebral infarcts [6], while TWiTCH established hydroxycarbamide as a non‐inferior alternative for maintaining transcranial Doppler velocities in selected patients [7]. Current ASFA and ASH guidelines recommend chronic RCE for primary and secondary stroke prevention, recurrent acute chest syndrome, and selected patients with frequent vaso‐occlusive crises [8, 9].

Automated RCE removes the patient's sickle erythrocytes and replaces them with donor HbA‐containing red cells, diluting the circulating HbS fraction. The therapeutic goal is to maintain the pre‐procedure HbS below a defined threshold, typically under 30% for stroke prevention or under 50% for other indications, while achieving a post‐procedure hematocrit of approximately 30%, targets supported by ASH 2020 transfusion guidance and reinforced by subsequent expert reviews [9, 10]. Compared with simple transfusion, automated RCE offers improved iron balance and more predictable HbS reduction per procedure [10, 11]. The fraction of cells remaining (FCR) is the key procedural parameter, optimized to the available RBC units and desired HbS target [12, 13].

Despite these advances, scheduling of chronic RCE remains largely empirical. Standard practice relies on uniform intervals of every 3–6 weeks, adjusted by trial and error as patient‐specific data accumulate [14]. This approach does not account for inter‐patient variability in HbS rebound kinetics. Patients with rapid rebound may exceed their therapeutic threshold well before their next scheduled procedure, while patients with slow kinetics may undergo unnecessarily frequent procedures, incurring excess procedural burden and blood‐product use without commensurate benefit.

Rogers et al. [14] recently introduced the daily rate of rise (ROR) in HbS as a quantitative metric to characterize individual HbS rebound kinetics. In their retrospective analysis of 660 procedures from 24 patients, they demonstrated that the ROR is a relatively stable, patient‐specific parameter that is not significantly influenced by changes in post‐procedure hematocrit or concurrent hydroxycarbamide use. Using a rolling three‐procedure average, the ROR predicted subsequent pre‐procedure HbS levels with moderate accuracy (*R* ^2^ = 0.65). Critically, however, their study was descriptive: it established the ROR as a clinically meaningful metric but did not translate it into a prescriptive scheduling algorithm.

In the present study, we extend the work of Rogers et al. [14] by developing a personalized, ROR‐driven scheduling formula for chronic RCE that integrates patient‐specific HbS kinetics with an adjustable safety factor for risk stratification. Our primary aims were to characterize ROR heterogeneity and intra‐patient stability in an independent adult cohort, to derive and evaluate a formula‐based interval prediction model, and to demonstrate how an adjustable safety factor enables risk‐adjusted scheduling. By risk‐adjusted scheduling we mean titrating the procedure interval to keep a laboratory parameter, the pre‐procedure HbS, within a target range, in the same spirit that other therapies are titrated to a therapeutic laboratory target.

## 2 Materials and Methods

### 2.1 Study Design and Patient Population

This was a retrospective, single‐center cohort study of adult patients with SCD undergoing chronic prophylactic RCE at Jackson Memorial Hospital. All procedures were performed in the outpatient apheresis setting in clinically stable patients. No inpatient procedures and no urgent or acute‐crisis exchanges (e.g., for acute chest syndrome, splenic sequestration, or acute vaso‐occlusive crisis) were included, so the analyzed kinetics reflect a stable steady state rather than an acutely perturbed one. The study was approved by the Institutional Review Board with a waiver of informed consent given its retrospective design (IRB# 20201331).

Patients were included if they had a confirmed diagnosis of SCD, were at least 18 years of age at first RCE, and had undergone at least five consecutive automated RCE procedures. Patients with fewer than five procedures, substantial missing laboratory data, or age under 18 at first RCE were excluded. No simple top‐up transfusions that could have affected ROR calculations were administered during the analyzed intervals. Hydroxyurea use was intermittent across the cohort and could not be reliably ascertained per encounter; it was therefore not used as an inclusion criterion or covariate, consistent with the finding of Rogers et al. [14] that ROR is not significantly influenced by concurrent hydroxycarbamide. The study period spanned July 2022 through November 2025.

A total of 28 patients were identified from institutional records. Of these, seven were excluded for limited data (fewer than five visits or missing values) and two were excluded as pediatric patients (under 18 years at first RCE), yielding 19 adult patients. Because there are no evidence‐based HbS targets in HbSC disease and the combined HbS + HbC fraction rather than HbS alone governs management, the single HbSC patient was removed from the pooled analyses and is reported separately. The primary analytic cohort therefore comprised 18 adult HbSS patients. Clinical indications for chronic RCE included primary and secondary stroke prevention, recurrent vaso‐occlusive crisis prevention, and acute chest syndrome prevention, consistent with ASFA [8] and ASH [9] guidance.

### 2.2 Current Scheduling Practice

To interpret the comparison between model‐recommended and actual intervals, current practice at our center is described here. Patients are assigned an empirical target interval of approximately every 4–5 weeks. This target is a uniform heuristic and is not individualized to a patient's HbS rebound kinetics. Patients then book procedures around that target subject to clinic capacity, appointment availability, and personal factors, so the realized interval for a given patient varies around the nominal target rather than following a strict fixed schedule. We use the term uniform empirical scheduling to describe this practice. Because the target itself is not derived from patient kinetics, deviations of the actual interval from the target reflect a combination of operational barriers and the absence of a principled, patient‐specific target.

### 2.3 Data Collection

Per‐encounter variables extracted included procedure date, RBC units exchanged, target and achieved FCR (%), target and achieved post‐procedure Hct (%), pre‐ and post‐procedure HbS (%), and serum ferritin. Patient‐level variables included age at chronic‐RCE initiation, sex, SCD genotype, and clinical indication. Hydroxyurea status was not reliably ascertainable per encounter and was not analyzed (see above).

### 2.4 RBC Exchange Procedure

All automated RCE procedures used the Spectra Optia apheresis system (Terumo BCT, Lakewood, CO). The target FCR was individualized to available RBC units and clinical status, generally 30%–50% [12, 13]; the standard post‐procedure Hct target was 30% [14]. Both isovolemic exchange and depletion/exchange protocols were used and were not separated for analysis. Donor RBC units were antigen‐matched per institutional alloimmunization prevention protocols [10, 15]. HbS was determined by high‐performance liquid chromatography as part of routine clinical care [16].

### 2.5 ROR Calculation

The daily ROR in HbS was calculated for each inter‐procedure interval as described by Rogers et al. [14]:

$$\begin{array}{c} \mathrm{ROR}=\left(\mathrm{Pre}-\text{procedure}\;\mathrm{HbS}\%-\text{Prior post}-\text{procedure}\;\mathrm{HbS}\%\right)\\ \;/\text{Interval}\;\left(\text{days}\right) \end{array}$$

For each patient, a personal ROR was derived by averaging all calculable inter‐procedure ROR values. Unlike Rogers et al. [14], who restricted analysis to intervals of 42 days or fewer to avoid decay‐phase confounding, we included all available outpatient intervals to reflect real‐world scheduling variability. Intervals involving inpatient exchanges (two procedures in a single patient, one of which produced a prolonged 215‐day gap) were excluded so that the analysis reflects stable outpatient kinetics. First visits, which lacked a prior post‐procedure HbS value, did not yield a calculable interval.

Two complementary metrics were also computed. The rate of fall (ROF) in hematocrit was calculated as the daily decline in Hct between procedures:

$$\begin{array}{c} \mathrm{ROF}=\left(\text{Post}-\text{procedure}\;\mathrm{Hct}\%-\text{Next}\;\mathrm{pre}-\text{procedure}\;\mathrm{Hct}\%\right)\\ \;/\text{Interval}\;\left(\text{days}\right) \end{array}$$

The S‐production index (SPI) was defined as the ratio ROR/ROF, providing a composite measure of how efficiently HbS‐containing erythrocytes accumulate relative to overall RBC turnover. Because every procedure targeted a post‐procedure Hct of about 30%, the starting point of each rebound interval was standardized; the residual variation in how far Hct falls before the next exchange is captured by ROF, and therefore by SPI. A patient who falls further between procedures (lower pre‐procedure Hct) has a higher ROF and a lower SPI. A high SPI (ROF near zero) indicates a stable hematocrit with progressive HbS accumulation, while a low SPI indicates that Hct decline and HbS rise occur in parallel.

### 2.6 Personalized Interval Model

Building on the descriptive work of Rogers et al. [14], who showed that individual ROR values are stable and predictive but did not translate them into a scheduling algorithm, we derived a formula to estimate the optimal interval between RCE procedures:

$$t_{\text{interval}}=S_{\text{risk}}\times \left({\mathrm{HbS}}_{\text{threshold}}-{\mathrm{HbS}}_{\text{post}}\right)/{\mathrm{ROR}}_{\text{personal}}$$

where HbS_threshold_ is the pre‐exchange target (30% for stroke prevention or 50% for other indications) [7, 17], HbS_post_ is the achieved post‐exchange HbS%, ROR_personal_ is the patient's average daily rate of rise, and *S*_risk_ is an adjustable safety factor (0.80–1.0) used to tighten intervals for higher‐risk patients [5, 6]. Notably, the formula has two manipulable inputs: ROR, which reflects fixed patient biology, and HbS_post_, which is modifiable through the procedural FCR and exchange volume. The model was applied retrospectively to compare model‐recommended intervals with actual scheduling.

### 2.7 Statistical Analysis

Descriptive statistics are reported as mean ± SD and range. Intra‐patient ROR consistency was assessed by coefficient of variation (CV%). Predictive accuracy was evaluated by comparing predicted versus observed pre‐procedure HbS using the patient's overall mean ROR; a rolling three‐procedure average ROR was also tested. Model performance was quantified by *R* ^2^, RMSE, and mean prediction bias. The relationship between pre‐procedure Hct and the SPI and ROR was examined by correlation. SPI was undefined for patients whose mean ROF was negative (a stable or rising inter‐procedure hematocrit), since a negative denominator makes continuous SPI comparison meaningless; these patients were excluded from SPI analyses. Because the SPI distribution was right‐skewed and sensitive to a single high value, the pre‐procedure Hct versus SPI association is reported primarily by the rank‐based Spearman correlation, which is robust to outliers, with the Pearson coefficient given alongside. Prespecified sensitivity analyses included exclusion of inpatient exchanges and the associated 215‐day interval, separate reporting of the HbSC patient, a subgroup analysis restricted to patients with CV below 20%, and exclusion of each patient's first interval. Analyses used Python 3.10 with NumPy and SciPy.

## 3 Results

### 3.1 Patient and Procedure Characteristics

Eighteen adult HbSS patients undergoing chronic prophylactic outpatient RCE comprised the primary cohort; the single HbSC patient is reported separately below. The 18 patients contributed a median of 6 procedures each (range 5–8), yielding 107 analyzed outpatient procedures and 87 calculable inter‐procedure intervals after excluding first visits, inpatient exchanges, and the single 215‐day gap, over a median follow‐up of 7.4 months (range 5.1–13.3 months). Clinical indications included primary and secondary stroke prevention, recurrent vaso‐occlusive crisis prevention, and acute chest syndrome prevention.

Procedure‐level characteristics are summarized in Table 1. The mean number of RBC units exchanged per procedure was 5.8 ± 1.1 (range 3–8). The mean achieved FCR was 39.7% ± 5.8%, closely matching the mean target FCR of 40.4% ± 5.4%. Pre‐procedure HbS averaged 42.8% ± 9.9% (range 24.8%–66.3%), reflecting wide inter‐patient variability in rebound kinetics and scheduling. Post‐procedure HbS averaged 15.5% ± 4.2% (range 8.3%–33.6%). After excluding inpatient exchanges and the associated 215‐day gap, the mean inter‐procedure interval was 45.0 ± 12.1 days (median 42; range 23–84). One serum ferritin value originally recorded as 74 410 ng/mL was identified as a transcription error and corrected to 7441 ng/mL; the highest accurate value was 15 368 ng/mL, consistent with transfusional iron burden in a chronically transfused patient. Serum ferritin did not enter the ROR, ROF, SPI, or interval calculations, so it does not affect the kinetic estimates.

**TABLE 1:** Procedure‐level characteristics.

Parameter	*N*	Mean ± SD	Median	Range	IQR
RBC units/procedure	106	5.8 ± 1.1	6.0	3–8	5–7
Target FCR (%)	107	40.4 ± 5.4	40.0	30–55	40–40
Actual FCR (%)	107	39.7 ± 5.8	40.0	28–57	36–42
Pre‐procedure HbS (%)	107	42.8 ± 9.9	43.4	24.8–66.3	34.4–49.8
Post‐procedure HbS (%)	107	15.5 ± 4.2	14.9	8.3–33.6	12.7–17.5
Pre‐procedure Hct (%)	107	25.9 ± 4.0	24.7	19.1–36.8	23.1–28.1
Post‐procedure Hct (%)	107	30.0 ± 1.9	29.9	25.3–35.0	28.9–31.1
Inter‐procedure interval (days)	87	45.0 ± 12.1	42	23–84	36–50
Ferritin (ng/mL)	95	4029 ± 3574	3030	18–15 368	940–6186

*Note:* Values reflect the 107 outpatient procedures contributed by the 18 HbSS patients; the single HbSC patient is reported separately.

Abbreviations: FCR = fraction of cells remaining, HbS = hemoglobin S, Hct = hematocrit.

### 3.2 ROR: Distribution and Heterogeneity

**FIGURE 1:**
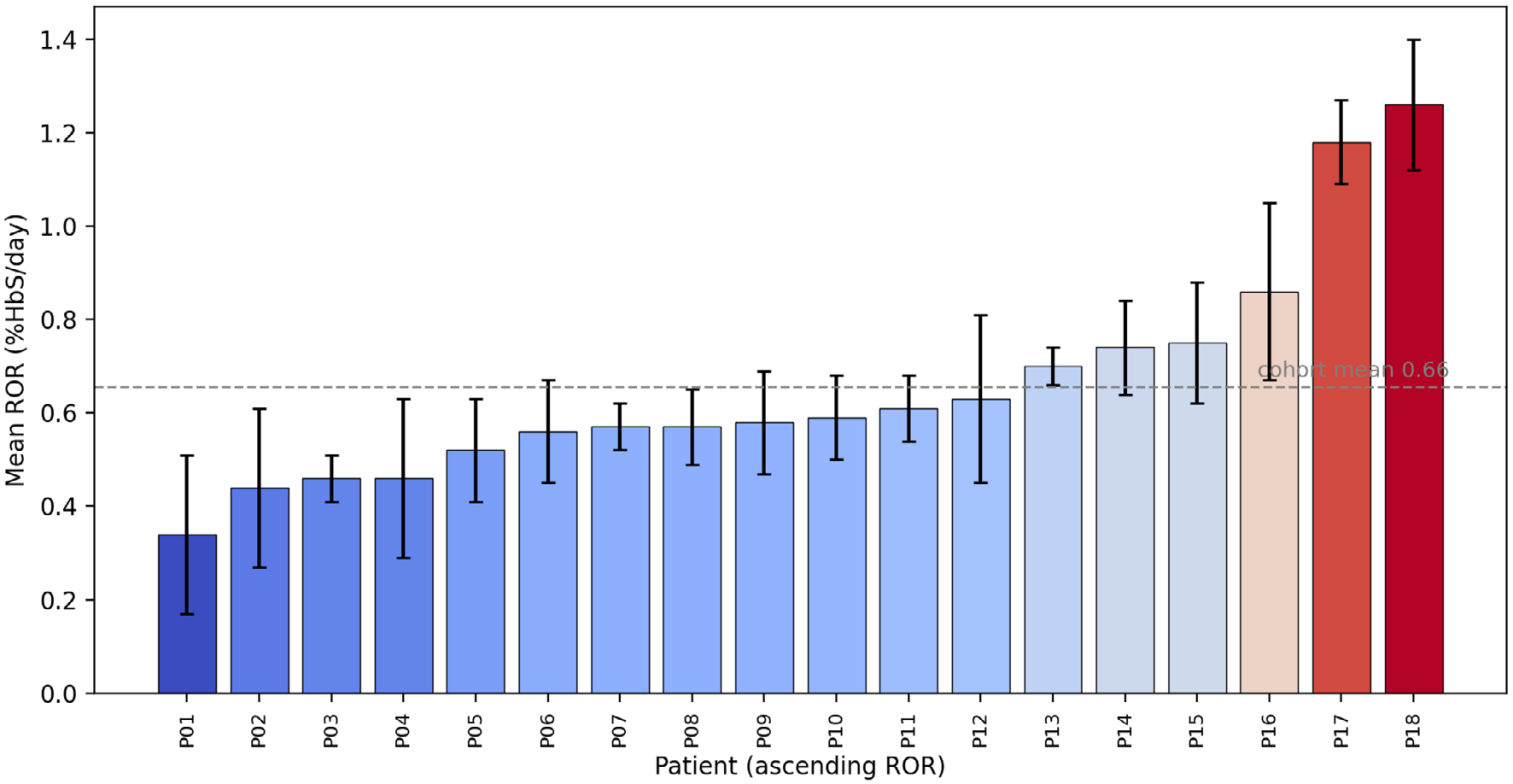
Per‐patient mean rate of rise (ROR) in hemoglobin S. Bars represent the mean ROR for each HbSS patient ordered by ascending value, with error bars indicating one standard deviation. The dashed line marks the cohort mean ROR of 0.66%HbS/day. Bar color transitions from blue (slow rebounders) to red (fast rebounders). A 3.7‐fold difference is observed between the slowest (Patient 01, 0.34%/day) and fastest (Patient 18, 1.26%/day) rebounders.

Across the HbSS cohort, the mean patient‐level ROR was 0.66%HbS/day ± 0.24%HbS/day (median 0.58; range 0.34–1.26; IQR 0.53–0.73; Figure 1; Table 2). This represents a 3.7‐fold difference between the slowest and fastest rebounders, underscoring the substantial inter‐patient heterogeneity in HbS rebound kinetics previously reported by Rogers et al. [14]. Four patients had a slow ROR (under 0.50%/day), 12 had a moderate ROR (0.50%/day–1.00%/day), and two had a rapid ROR (1.00%/day or higher). Cross‐validation of patient‐level ROR values derived independently from encounter‐level calculations confirmed agreement with summary statistics.

**TABLE 2:** Per‐patient rate of rise, rate of fall, S‐Production index, and model‐predicted versus actual intervals (HbSS cohort).

Patient	*N*	ROR	SD	CV%	ROF	SPI	Model	Actual mean	Actual IQR
Patient 01	6	0.34	0.17	51.5	0.128	2.65	42.8	65.8	58–77
Patient 02	6	0.44	0.17	38.0	0.118	3.74	38.5	47.4	43–56
Patient 03	6	0.46	0.05	11.3	0.081	5.64	30.1	57.0	54–62
Patient 04	5	0.46	0.17	36.6	0.169	2.70	37.9	50.5	49–53
Patient 05	6	0.52	0.11	20.5	0.129	3.99	30.1	44.4	35–42
Patient 06	6	0.56	0.11	20.5	0.107	5.21	32.9	52.2	47–51
Patient 07	6	0.57	0.05	8.5	0.012	49.11	15.4	53.2	43–56
Patient 08	6	0.57	0.08	14.0	0.128	4.46	32.2	53.5	51–56
Patient 09	6	0.58	0.11	18.3	0.117	4.93	25.9	37.6	35–37
Patient 10	6	0.59	0.09	14.8	0.143	4.10	27.1	45.0	42–49
Patient 11	6	0.61	0.07	11.7	0.120	5.10	29.6	40.8	42–42
Patient 12	6	0.63	0.18	28.3	0.068	9.38	30.4	40.8	39–42
Patient 13	6	0.70	0.04	5.8	0.174	4.04	18.4	45.0	42–44
Patient 14	6	0.74	0.10	14.0	−0.057	n/d	17.4	49.2	42–50
Patient 15	6	0.75	0.13	17.1	0.083	9.06	17.3	46.2	49–49
Patient 16	8	0.86	0.19	21.8	−0.005	n/d	12.5	30.9	27–36
Patient 17	6	1.18	0.09	7.8	−0.020	n/d	12.8	33.6	35–35
Patient 18	6	1.26	0.14	11.2	0.138	9.16	6.3	30.8	28–35

*Note:* Patients ordered by ascending ROR. Model interval = (30% − mean post‐HbS%)/ROR at *S*(risk) = 1.0. Actual mean and IQR are observed inter‐procedure intervals (days). *N* is the number of procedures each patient contributed; for Patient 08, two inpatient exchanges were excluded, so that patient's ROR, CV, and SPI derive from two outpatient intervals, and interquartile ranges based on fewer than three intervals should be interpreted with caution. The Patient 18 model interval of 6 days is retained as computed; it reflects that patient's shallow exchanges (mean post‐HbS 22%) rather than a data error and is discussed under FCR as an alternative lever.

Abbreviations: CV = coefficient of variation, ROF = rate of fall (%Hct/day), ROR = rate of rise (%HbS/day), SD = standard deviation, SPI = S‐production index (ROR/ROF) (reported as n/d where mean ROF was nonpositive).

### 3.3 Intra‐Patient ROR Stability

Within‐patient ROR consistency was assessed using the CV%. The median intra‐patient CV was 16.0% (mean 19.5%; range 5.8%–51.5%). The majority of patients (11 of 18, 61%, with CV below 20%) demonstrated a highly reproducible ROR across procedures, and this subgroup is where the model is most defensible. Two patients (Patient 01 and Patient 02) showed higher variability (CV over 38%), both among the slowest rebounders (0.34%/day and 0.44%/day); in slow rebounders, small absolute HbS changes inflate the relative CV, so high CV in this subgroup should be interpreted with that in mind. Conversely, the two fastest rebounders (Patient 17 and Patient 18, ROR 1.18%/day and 1.26%/day) had low CVs (7.8% and 11.2%), suggesting that rapid rebounders exhibit particularly consistent kinetics. These observations support using demonstrated ROR stability, for example a CV below 20% established over at least three procedures, as a gating criterion before applying model‐based scheduling.

### 3.4 ROF, SPI, and Pre‐Procedure Hematocrit

The mean ROF across the cohort was 0.09%Hct/day ± 0.07%Hct/day (range −0.06 to 0.17). Three patients (Patient 14, Patient 16, and Patient 17) had a negative mean ROF (Table 2), reflecting a stable or rising inter‐procedure hematocrit. Because a negative ROF produces a negative SPI regardless of ROR magnitude, continuous SPI comparison is meaningless for these patients; SPI was therefore undefined for them, and they were excluded from SPI analyses. This negative‐ROF pattern may reflect endogenous erythropoiesis offsetting the expected post‐exchange hematocrit decline, although erythropoietin and reticulocyte data were not available. Among the 15 patients with a defined SPI, the distribution was right‐skewed with a median of 4.9 (range 2.65–49.1).

Consistent with the design of these metrics, patients with lower pre‐procedure Hct (those who fell further between exchanges) showed a higher ROF (pre‐procedure Hct vs. ROF *r* = −0.94, *p* < 0.001) and, among patients with a defined SPI, a correspondingly lower SPI (pre‐procedure Hct vs. SPI Spearman rho = +0.75, *p* = 0.001; Pearson *r* = +0.82). This indicates that SPI, rather than ROR alone, distinguishes HbS rising predominantly from new production (high SPI, stable Hct) from HbS rising alongside general RBC turnover (low SPI, falling Hct), addressing the influence of the starting hematocrit on apparent rebound.

### 3.5 HbSC Patient (Reported Separately)

The single HbSC patient is described separately because transfusion targets in HbSC disease are not evidence‐based and management is generally directed at the combined HbS + HbC fraction rather than HbS alone. In this single HbSC patient, the ROR was 0.58%HbS/day (CV 9.4%), ROF 0.04%Hct/day, SPI 13.5, mean post‐procedure HbS 6.4%, and mean observed interval 29 days (median 28), with a model‐recommended interval of approximately 40 days. While HbS rebound is expected to correlate with HbC rebound and the kinetic approach may still be meaningful, an HbS‐only metric may not fully capture the relevant burden, so this case should be interpreted with caution and is excluded from the pooled HbSS results.

### 3.6 Predictive Accuracy of the ROR‐Based Model

Using each patient's overall mean ROR and the formula HbS(predicted) = HbS(post) + (ROR × interval), predicted pre‐procedure HbS values were compared with observed values across all calculable intervals (*n* = 87). The model achieved *R* ^2^ = 0.72, RMSE = 5.2%HbS, and mean bias +0.2%HbS. This improves on the originally submitted 19‐patient analysis (*R* ^2^ = 0.46 across 96 intervals): restricting the cohort to the 18 HbSS outpatient patients and excluding inpatient exchanges and the 215‐day interval removed the highest‐residual points, raising the fit without any change to the method. A rolling three‐procedure average ROR did not improve accuracy over the overall patient mean, consistent with the limited encounter count per patient (median 6). Because our estimate applies each patient's overall mean ROR within sample, it is not directly comparable to the rolling, forward‐looking *R* ^2^ of 0.65 reported by Rogers et al. [14]; we therefore present it as a descriptive within‐cohort fit rather than as evidence of superior prediction.

### 3.7 Model‐Recommended vs. Actual Scheduling

**FIGURE 2:**
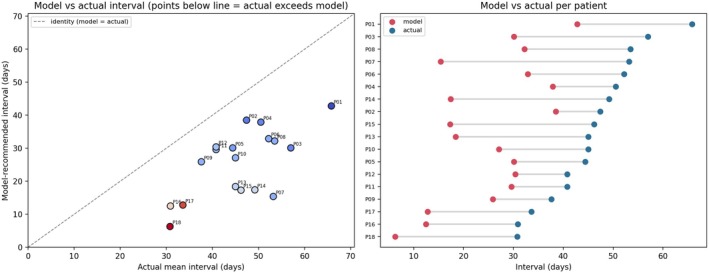
Comparison of model‐recommended versus actual scheduling intervals. Left: Scatter plot of model‐recommended interval (*S*(risk) = 1.0, HbS threshold = 30%) against actual mean interval for each patient. Points below the identity line indicate patients whose actual scheduling exceeded the model‐recommended interval. Right: Dumbbell chart showing the discrepancy for each patient, with one marker for the model‐recommended interval and one for the actual mean interval.

Applying the interval formula with *S*(risk) = 1.0 and HbS(threshold) = 30%, the model recommended substantially shorter intervals than those actually used for the majority of patients (Figure 2; Table 2). Because current practice assigns a uniform empirical target of every 4–5 weeks that is not individualized to kinetics, this indicates that the prevailing target is not aligned with patient‐specific rebound. Per‐patient interval variability (Table 2, Actual IQR) shows that realized intervals also vary around the target; for example, one patient's mean interval of 66 days is well beyond the 4 to 5 week target, reflecting scheduling and access barriers rather than a prescribed plan. The two fastest rebounders (Patient 17, ROR 1.18; Patient 18, ROR 1.26) were scheduled at mean intervals of 34 and 31 days while the model predicted they would reach the 30% threshold within roughly 13 days and 6 days, respectively, spending most of each interval above the surrogate target. The slowest rebounders (Patient 01, ROR 0.34; Patient 02, ROR 0.44) had model‐predicted intervals of 43 and 39 days, suggesting safe extension is feasible. No acute clinical events (stroke or vaso‐occlusive crisis requiring escalation) occurred in any patient during the study window.

### 3.8 Clinical Scenario Modeling

To illustrate the formula's utility, a slow rebounder (ROR = 0.34%/day, post‐HbS = 10%, threshold = 30%) is predicted an interval of 59 days at *S*(risk) = 1.0 (50 days at *S*(risk) = 0.85), supporting 6–7 week scheduling rather than 4 weeks and an estimated reduction of 10–15 RBC units per year for that patient (cohort mean of 5.8 units per procedure times the projected reduction in annual procedure count). In contrast, a rapid rebounder (ROR = 1.26%/day) with a post‐HbS of 10% is predicted to reach the 30% threshold in about 16 days at *S*(risk) = 1.0 (about 13 days at *S*(risk) = 0.85). Importantly, post‐HbS is itself adjustable: a deeper exchange lowering post‐HbS to 5% extends the same rapid rebounder's predicted interval to about 20 days, while relaxing the pre‐procedure HbS threshold to 50% extends it to about 32 days, demonstrating that the formula accommodates both indication‐specific targets and procedural intensity while preserving patient‐specific kinetics.

## 4 Discussion

### 4.1 Summary of Key Findings

This study presents a personalized, ROR‐driven scheduling framework for chronic outpatient RCE in SCD. Across the HbSS cohort, we observed substantial inter‐patient heterogeneity in HbS rebound kinetics (ROR range 0.34%HbS/day–1.26%HbS/day, a 3.7‐fold difference), with within‐patient stability sufficient to support interval prediction in about half of patients (those with CV below 20%). Retrospective application demonstrated that most patients were scheduled at intervals longer than those predicted to maintain HbS below 30%, indicating that the uniform empirical target is not aligned with patient‐specific kinetics. Two complementary metrics, ROF and SPI, provided additional kinetic characterization and clarified the influence of the starting hematocrit on apparent rebound.

### 4.2 Comparison With Rogers Et Al. [14]

Our findings confirm and extend Rogers et al. [14]. We replicated their central observation that ROR is a stable, patient‐specific parameter, with a similar range and comparable intra‐patient consistency. Our patient mean ROR achieved a within‐sample *R* ^2^ = 0.72 for predicting pre‐procedure HbS, with minimal bias. Because Rogers et al. used a rolling, forward‐looking average (*R* ^2^ = 0.65) in a larger per‐patient series, the two values are not directly comparable; we interpret our fit as a descriptive indication that the overall mean ROR captures most of the within‐cohort variation, not as superior predictive performance. Where our study departs from Rogers et al. [14] is in translating the descriptive ROR into a prescriptive scheduling tool. Our formula provides a transparent, clinically actionable framework, and the adjustable safety factor adds a risk‐stratification dimension absent from prior descriptive work.

### 4.3 Clinical Implications and Net Blood Utilization

The discrepancy between model‐recommended and actual intervals indicates that under the current uniform target most patients spend substantial inter‐procedure time above their surrogate HbS target. This is a gap in HbS exposure relative to a guideline‐based threshold, not a demonstrated clinical gap: no stroke or vaso‐occlusive events occurred in this cohort, consistent with its small size and short follow‐up, and any outcome significance remains unproven.

A balanced accounting of blood utilization is essential. Under the model at *S*(risk) = 1.0 and a 30% threshold, only a minority of patients (the slow rebounders, roughly one fifth of the cohort) would qualify to extend intervals, with an estimated reduction of 10–15 donor RBC units annually each. The majority would instead require shorter intervals to remain below threshold. Consequently, if the model were applied to strictly maintain HbS below 30% in all patients, program‐level blood product utilization would likely increase rather than decrease. The value of the framework is therefore better described as improved precision and reallocation, directing resources toward patients with the largest target‐attainment gap and away from over‐treated slow rebounders, rather than as a guaranteed reduction in overall blood use. Whether the additional product use translates into better outcomes is precisely what prospective study must determine, and this trade‐off is central to weighing the model against blood stewardship goals.

### 4.4 FCR as an Alternative Lever

Shortening the interval is not the only response to a fast rebounder. The interval formula has two manipulable inputs: ROR, which is fixed biology, and post‐procedure HbS, which is set by the procedural FCR and exchange volume. Lowering the FCR (a deeper exchange) reduces post‐HbS and lengthens the achievable interval. For example, at ROR 1.26%/day, a post‐HbS of 15% versus 10% versus 5% shifts the predicted time to a 30% threshold from roughly 12 to 16 to 20 days. With a cohort mean FCR near 40% at about 6 units per procedure, there is headroom to intensify the exchange for selected rapid rebounders rather than resorting to impractically frequent visits. This strategy is constrained by RBC unit availability, cost, and venous access, and where deeper exchange is not feasible the interval must be shortened instead. Presenting both levers gives clinicians a realistic menu for real‐world implementation.

### 4.5 The Safety Factor as a Risk‐Stratification Tool

The adjustable safety factor accommodates clinical heterogeneity in risk tolerance. We propose *S*(risk) = 0.80–0.85 for a history of overt stroke or severe cerebral vasculopathy such as moyamoya syndrome; *S*(risk) = 0.90–0.95 for silent cerebral infarcts or abnormal transcranial Doppler velocities; and *S*(risk) = 1.0 for lower‐risk indications such as recurrent vaso‐occlusive crisis or acute chest syndrome prevention without cerebrovascular disease. The intended parallel is to titrating therapy toward a therapeutic laboratory target range; the safety factor simply scales how conservatively the interval is set. The appropriate *S*(risk) should ultimately be determined by prospective validation linking time‐above‐threshold to clinical outcomes.

### 4.6 ROF and SPI: Complementary Kinetic Markers

The ROF and SPI, reported in Table 2 as descriptive secondary metrics, extend the kinetic characterization of these patients and clarify the effect of the starting hematocrit. Because all post‐procedure Hct values were matched to about 30%, the degree of subsequent Hct decline (ROF) distinguishes patients in whom HbS rises chiefly from new production (high SPI, stable Hct) from those in whom HbS rises alongside general RBC turnover (low SPI, falling Hct). A small subset had a negative ROF (stable or rising hematocrit), for which SPI is undefined; these were reported descriptively and excluded from SPI analyses. These secondary metrics are exploratory and are not the focus of the present scheduling model.

### 4.7 Limitations

Several limitations should be acknowledged. This was a retrospective, single‐center study with a modest sample (HbSS adults only), which limits power for subgroup analyses and may not capture the full ROR variability across SCD populations. The single HbSC patient was analyzed separately because HbSC transfusion targets are not evidence‐based, and pediatric generalizability requires separate validation. Intra‐patient ROR variability was wide in some patients (CV up to 51.5%), and the model is most reliable in the subgroup with demonstrated kinetic stability; in high‐variability patients, a more conservative safety factor and continued empirical monitoring are warranted. The linear model assumes a constant rate of HbS rise; kinetics may instead be curvilinear, particularly at longer intervals. Because inclusion required at least five consecutive procedures, most analyzed intervals reflect established chronic RCE rather than the initial loading phase, during which kinetics may differ; a sensitivity analysis excluding each patient's first interval did not materially change the cohort mean ROR (0.66 vs. 0.66%/day). Hydroxyurea use was intermittent and not analyzed, and erythropoietin levels were not available. Exchange and depletion/exchange procedures were analyzed together. Finally, even under current practice patients do not consistently achieve the nominal target interval, so real‐world implementation of any interval scheme, model‐based or otherwise, depends on operational adherence and clinic capacity; the model sets a kinetically justified target but does not by itself solve these scheduling barriers. The model has not been prospectively validated, and retrospective comparison of recommended versus actual intervals does not establish that adherence to the recommendation would improve outcomes. Finally, after excluding inpatient exchanges, one patient's ROR rested on only two outpatient intervals, so that individual estimate is less precise than those based on five or more.

### 4.8 Future Directions

Prospective, multicenter validation is the essential next step, examining whether adherence to model‐recommended intervals improves clinical outcomes (time within therapeutic HbS range, stroke incidence, vaso‐occlusive events) relative to uniform empirical scheduling, and whether any increase in blood product use is justified by those outcomes. Integration of the formula into EHR‐based clinical decision support would enable real‐time recommendations and prospective data collection, including the FCR lever as an explicit option. Machine‐learning approaches incorporating additional variables (genotype, erythropoietin, reticulocyte kinetics) may further refine predictions. Dedicated validation in pediatric cohorts and during the pediatric‐to‐adult transition warrants specific attention.

## 5 Conclusions

The daily ROR in HbS is a patient‐specific, reproducible metric with substantial inter‐patient variability that has direct implications for scheduling chronic RCE in SCD. The proposed formula provides a transparent, individualized framework that integrates objective HbS kinetics with risk‐stratified safety factors, and it is most applicable to patients with stable, established kinetics. Retrospective application shows that uniform empirical scheduling is not aligned with patient‐specific rebound for most patients. Because the majority of patients would require shorter rather than longer intervals to maintain HbS below 30%, implementing the model to strictly maintain target would likely increase program‐level blood utilization; its benefit is therefore best understood as improved precision and reallocation, with procedural FCR offering an additional lever, rather than as a guaranteed reduction in blood use. The complementary metrics ROF and SPI extend kinetic phenotyping. Prospective validation in independent adult cohorts, and separate validation in pediatric populations, is warranted to determine whether this framework improves outcomes.

## Funding

The authors have nothing to report.

## Ethics Statement

This study was approved by the University of Miami Institutional Review Board with a waiver of informed consent (IRB# 20201331).

## Conflicts of Interest

The authors declare no conflicts of interest.

## Data Availability

The data that support the findings of this study are available from the corresponding author upon reasonable request.

## References

[1] GBD 2021 Sickle Cell Disease Collaborators , “Global, Regional, and National Prevalence and Mortality Burden of Sickle Cell Disease, 2000 to 2021: A Systematic Analysis From the Global Burden of Disease Study 2021,” Lancet Haematology 10, no. 8 (2023): e585–e599, 10.1016/S2352-3026(23)00118-7. 37331373 PMC10390339

[2] G. J. Kato , F. B. Piel , C. D. Reid , et al., “Sickle Cell Disease,” Nature Reviews. Disease Primers 4 (2018): 18010, 10.1038/nrdp.2018.10. 29542687

[3] K. L. Hassell , “Population Estimates of Sickle Cell Disease in the U.S.,” American Journal of Preventive Medicine 38, no. 4 (2010): S512–S521, 10.1016/j.amepre.2009.12.022. 20331952

[4] D. C. Rees , T. N. Williams , and M. T. Gladwin , “Sickle‐Cell Disease,” Lancet 376, no. 9757 (2010): 2018–2031, 10.1016/S0140-6736(10)61029-X. 21131035

[5] R. J. Adams , D. Brambilla , and STOP 2 Trial Investigators , “Discontinuing Prophylactic Transfusions Used to Prevent Stroke in Sickle Cell Disease,” New England Journal of Medicine 353, no. 26 (2005): 2769–2778, 10.1056/NEJMoa050460. 16382063

[6] M. R. DeBaun , M. Gordon , R. C. McKinstry , et al., “Controlled Trial of Transfusions for Silent Cerebral Infarcts in Sickle Cell Anemia,” New England Journal of Medicine 371, no. 8 (2014): 699–710, 10.1056/NEJMoa1401731. 25140956 PMC4195437

[7] R. E. Ware , B. R. Davis , W. H. Schultz , et al., “Hydroxycarbamide Versus Chronic Transfusion for Maintenance of Transcranial Doppler Flow Velocities in Children With Sickle Cell Anaemia (TWiTCH): A Multicentre, Open‐Label, Phase 3, Non‐Inferiority Trial,” Lancet 387, no. 10019 (2016): 661–670, 10.1016/S0140-6736(15)01041-8. 26670617 PMC5724392

[8] L. Connelly‐Smith , C. R. Alquist , M. Grzeskowiak , et al., “Guidelines on the Use of Therapeutic Apheresis in Clinical Practice: Evidence‐Based Approach From the Writing Committee of the American Society for Apheresis: The Ninth Special Issue,” Journal of Clinical Apheresis 38, no. 2 (2023): 77–278, 10.1002/jca.22043. 37017433

[9] S. T. Chou , M. Alsawas , R. M. Engmann , et al., “American Society of Hematology 2020 Guidelines for Sickle Cell Disease: Transfusion Support,” Blood Advances 4, no. 2 (2020): 327–355, 10.1182/bloodadvances.2019001143. 31985807 PMC6988392

[10] G. E. Linder and S. T. Chou , “Red Cell Transfusion and Alloimmunization in Sickle Cell Disease,” Haematologica 106, no. 7 (2021): 1805–1815, 10.3324/haematol.2020.270546. 33792218 PMC8252926

[11] C. D. Josephson , L. L. Su , K. L. Hillyer , and C. D. Hillyer , “Transfusion in the Patient With Sickle Cell Disease: A Critical Review of the Literature and Transfusion Guidelines,” Transfusion Medicine Reviews 21, no. 2 (2007): 118–133, 10.1016/j.tmrv.2006.11.003. 17397762

[12] J. Cid , M. Lozano , A. Fernandez , et al., “Theoretical and Practical Aspects of Fractional Cells Remaining in Automated Red Blood Cell Exchange With Spectra Optia,” Transfusion 55, no. 10 (2015): 2402–2408, 10.1111/trf.13042.

[13] K. R. Buban and C. E. Lawrence , “Algorithm‐Based Selection of Automated Red Blood Cell Exchange Procedure Goals Reduces Blood Utilization in Chronically Transfused Adults With Sickle Cell Disease,” Journal of Clinical Apheresis 37, no. 5 (2022): 468–475, 10.1002/jca.22004. 36053868

[14] K. Rogers , M. Alsawas , J. Chapman , A. J. Schlueter , and C. M. Knudson , “Using the Daily Rate of Rise in Hemoglobin S to Manage RBC Depletion/Exchange Treatment in Sickle Cell Disease,” Transfusion 64, no. 4 (2024): 685–692, 10.1111/trf.17797. 38506484

[15] S. T. Chou , P. Evans , S. Vege , et al., “RH Genotype Matching for Transfusion Support in Sickle Cell Disease,” Blood 132, no. 11 (2018): 1198–1207, 10.1182/blood-2018-05-851360. 30026182

[16] S. Van Aelst , H. Claerhout , E. Nackers , K. Desmet , and D. Kieffer , “Hemoglobin S Monitoring on TOSOH G8 in Hemoglobin A1c Mode in Case of Urgent Red Blood Cell Exchange,” Journal of Clinical Laboratory Analysis 32, no. 7 (2018): e22453, 10.1002/jcla.22453. 29667731 PMC6817183

[17] R. J. Adams , V. C. McKie , L. Hsu , et al., “Prevention of a First Stroke by Transfusions in Children With Sickle Cell Anemia and Abnormal Results on Transcranial Doppler Ultrasonography,” New England Journal of Medicine 339, no. 1 (1998): 5–11, 10.1056/NEJM199807023390102. 9647873

